# Effects of acute administration of trimethylamine N-oxide on endothelial function: a translational study

**DOI:** 10.1038/s41598-022-12720-5

**Published:** 2022-05-23

**Authors:** Anne Jomard, Luca Liberale, Petia Doytcheva, Martin F. Reiner, Daniel Müller, Michele Visentin, Marco Bueter, Thomas F. Lüscher, Roberto Vettor, Thomas A. Lutz, Giovanni G. Camici, Elena Osto

**Affiliations:** 1 Laboratory for Translational Nutrition Biology, ETH Zurich, Zurich, Switzerland; 2 Institute of Clinical Chemistry, University Hospital Zurich, Wagistrasse, 14, 8952 Schlieren, Switzerland; 3 Centre for Molecular Cardiology, University of Zurich, Zurich, Switzerland; 4 First Clinic of Internal Medicine, Department of Internal Medicine, University of Genoa, Genoa, Italy; 5 Institute of Veterinary Physiology, University of Zurich, Zurich, Switzerland; 6 Department of Clinical Pharmacology and Toxicology, University and University Hospital Zurich, Zurich, Switzerland; 7 Department of Surgery and Transplantation, University Hospital Zurich, Zurich, Switzerland; 8 Department of Medicine, University of Padova, Via Giustiniani, 2, 35128 Padua, Italy; 9 Department of Cardiology, Heart Center, University Hospital Zurich, Zurich, Switzerland

**Keywords:** Physiology, Cardiology, Molecular medicine

## Abstract

Elevated circulating levels of nutrient-derived trimethylamine N-oxide (TMAO) have been associated with the onset and progression of cardiovascular disease by promoting athero-thrombosis. However, in conditions like bariatric surgery (Roux-en-Y gastric bypass, RYGB), stable increases of plasma TMAO are associated with improved endothelial function and reduced cardiovascular morbidity and mortality, thus questioning whether a mechanistic relationship between TMAO and endothelial dysfunction exists. Herein, we translationally assessed the effects of acute TMAO exposure on endothelial dysfunction, thrombosis and stroke. After RYGB, fasting circulating levels of TMAO increased in patients and obese rats, in parallel with an improved gluco-lipid profile and higher circulating bile acids. The latter enhanced FXR-dependent signalling in rat livers, which may lead to higher TMAO synthesis post RYGB. In lean rats, acute TMAO injection (7 mg kg^−1^) 1.5-h before sacrifice and ex-vivo 30-min incubation of thoracic aortas with 10^−6^ M TMAO did not impair vasodilation in response to acetylcholine (Ach), glucagon-like peptide 1, or insulin. Similarly, in lean WT mice (n = 5–6), TMAO injection prior to subjecting mice to ischemic stroke or arterial thrombosis did not increase its severity compared to vehicle treated mice. Endothelial nitric oxide synthase (eNOS) activity and intracellular stress-activated pathways remained unaltered in aorta of TMAO-injected rats, as assessed by Western Blot. Pre-incubation of human aortic endothelial cells with TMAO (10^−6^ M) did not alter NO release in response to Ach. Our results indicate that increased plasmatic TMAO in the near-physiological range seems to be a neutral bystander to vascular function as translationally seen in patients after bariatric surgery or in healthy lean rodent models and in endothelial cells exposed acutely to TMAO.

## Introduction

The rising prevalence of overweight, obesity and cardio-metabolic disease highlights the need to understand better the complex interactions between the metabolic state and cardiovascular health. Meat and dairy derived L-carnitine, phosphatidylcholine and choline are catabolized by the gut microbiome into the volatile compound Trimethylamine (TMA). Through the portal vein, TMA reaches the liver where it is oxidised to Trimethylamine N-oxide (TMAO) by members of the flavin-containing monooxygenases (FMO) family, in particular isoform 3 (FMO3)^1^ under the control of nuclear receptor farnesoid-X-receptor (FXR)^1^. Previous studies indicated that TMAO and its precursors may be correlated to the onset and progression of athero-thrombotic cardiovascular disease^2,3^ through promotion of pathologic changes including cardiac and kidney fibrosis^4,5^, enhanced platelet reactivity and thrombus formation^6^, tight junctions disruption^7^, NLRP3 inflammasome activation and senescence of endothelial cells^8^. However, some evidence argues against a causative role of TMAO in cardiovascular disease. Fish consumption, which is a well-known protective factor against coronary heart disease and stroke^9^, also increases circulating TMAO^10^. Further, TMAO levels were not increased nor associated with atherosclerotic disease-risk in patients with already established atherosclerosis^11^. Indeed, very low (i.e. < 2 μmol/L) circulating TMAO was associated with higher mortality risk in deep vein thrombosis patients with the lowest risk of death observed for TMAO around 4 μmol/L^12^. Interestingly, higher concentrations of plasmatic TMAO (around 10 μmol/L) are reported^13^ after bariatric surgery, including Roux-en-Y Gastric Bypass (RYGB)^14^, which significantly decreases cardiovascular morbidity and mortality^15^ through beneficial improvement of obesity-induced endothelial dysfunction and inflammation^16,17^. Thus, the link between TMAO levels and different patho-physiological states, such as atherosclerosis, obesity and aging remain controversial. Further, cut-off levels for physiological versus pathological TMAO concentrations are difficult to define and there are limited data to assess whether modulation of TMAO production may translate into a therapeutic approach to reduce atherosclerosis risk. Based on our previous experimental results showing an improvement of obesity-induced endothelial dysfunction after RYGB^18^, which is reported to alter circulating levels of TMAO^14^, we investigated in this study whether acute TMAO administration was associated with endothelial dysfunction in rats or enhanced carotid thrombotic activity and stroke severity in mice.

## Results

### TMAO circulating levels increase in patients and rats after RYGB

**Figure 1:**
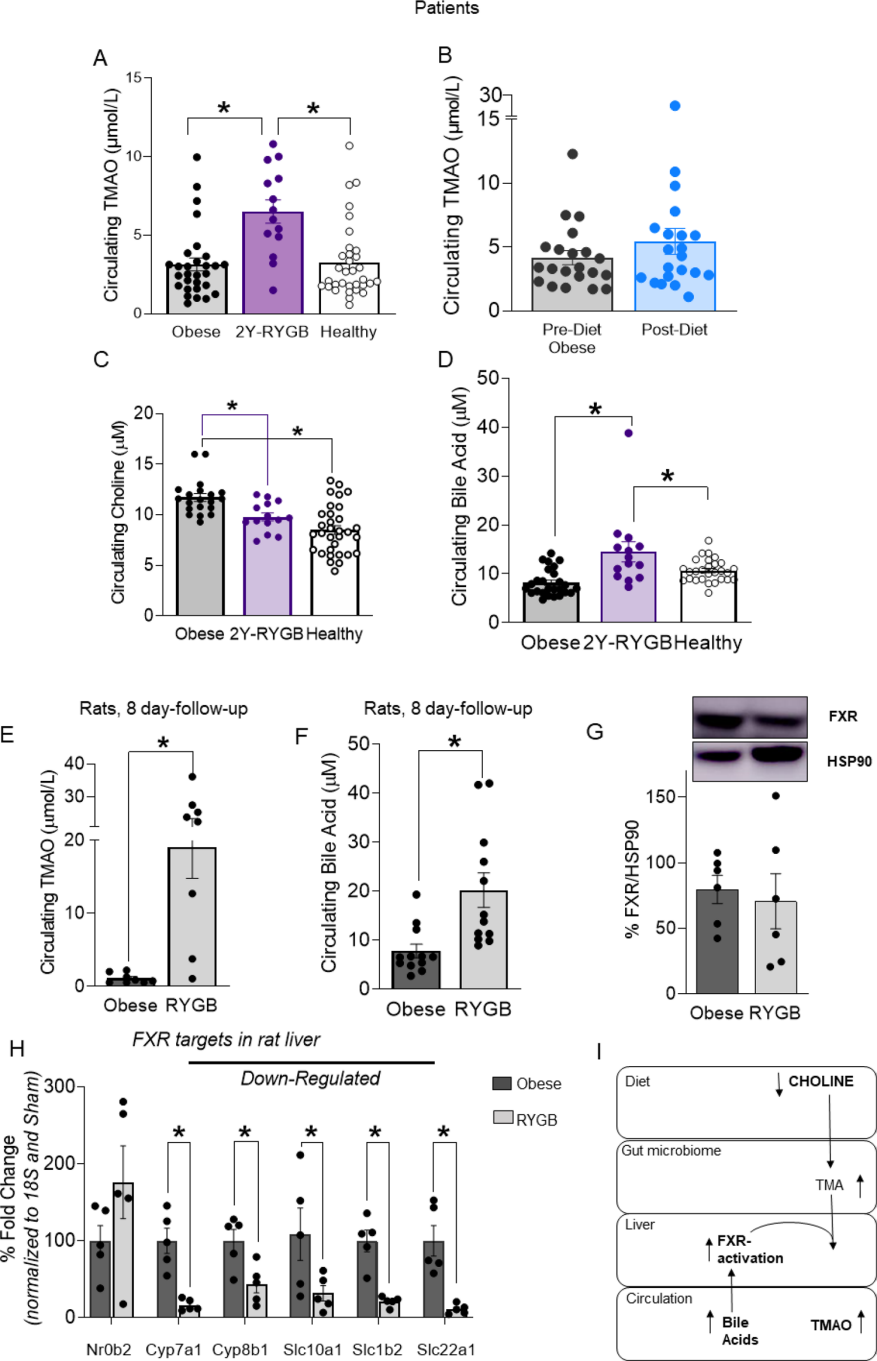
Circulating TMAO levels increase after bariatric surgery in patients and rats. Circulating TMAO levels in patients, (**A**) post-RYGB, (**B**) post-diet. (**C**) Circulating levels of the TMAO precursor choline, and (**D**) bile acids, which are upstream of TMAO synthesis (**I**). In rats 8-days post-RYGB or sham-surgery (obese), increase in (**E**) TMAO and (**F**) bile acids. In rat liver, (**G**) FXR protein expression (**H**) mRNA expression of target genes of FXR, i.e. Nr0b2 (encoding Shp protein), Cyp7a1, Cyp8b1, Slc10a1 (encoding Ntcp), Slc1b2 (encoding Oatp1b3), Slc22a1 (encoding Oct1)) in RYGB compared to obese sham-operated rats. (**I**) Diagram of TMAO synthesis and catabolism, targets in bold were measured in this study. Human data: RYGB patients n = 29–14, diet group n = 21, healthy subjects n = 33, 1-way ANOVA, Dunnett Correction. Rat data: n = 5–12. Original blots/gels of (**G**) are presented in Supplementary Fig. 4. Unpaired T-test. RYGB: Roux-en-Y Gastric Bypass, TMAO: trimethylamine N-oxide, TMA: trimethylamine, FXR: farnesoid-X-receptor. *p < 0.05.

TMAO levels in 29 obese patients before RYGB or diet treatment were similar to those of healthy controls (mean TMAO concentrations 3.13; 4.16 and 3.26 μmol/L, respectively, *p* = ns) (Fig. 1A,B). Circulating TMAO levels increased in patients 2-years after RYGB compared to baseline (6.50 vs 3.13 μmol/L, respectively, *p* < 0.05 Fig. 1A) but not in patients receiving a hypocaloric diet for an average duration of 19 months (Fig. 1B), suggesting that TMAO levels may increase as a consequence of RYGB-specific changes. We did not observe sex-specific differences in circulating TMAO levels neither in the RYGB or diet group (SOM Fig. 1A,B), nor in healthy subjects (TMAO female: 3.34 vs male: 3.31 μmol/L, *p* = ns). Baseline body weight (BW) was higher in obese candidates to RYGB compared to diet, as detailed in Table 1.

**Table 1:** Clinical characteristics of study populations.

	Pre-op obese	2-year-post-RYGB	Pre-diet obese	Post-diet	Healthy controls
Age (years)	40.6 ± 8.7	46.1 ± 6.7^a^	38.8 ± 13.9	40.3 ± 14.0	33.8 ± 9.9
N (M)	29 (9)	14 (6)	21 (6)	21 (6)	33 (14)
Weight (kg)	131.4 ± 19.6^a,c,d,e^	97.2 ± 15.5^a,b,d^	114.2 ± 21.7^a,b,c,e^	90.0 ± 13.9^a,d^	66.4 ± 11.1
BMI (kg m^−2^)	45.2 ± 5.7^a,d^	32.9 ± 4.1^a,b,d^	39.4 ± 7.2^a,b^	31.2 ± 4.2^a,b,e^	22.1 ± 2.1
Triglycerides (mmol L^−1^)	1.8 ± 1.0^a,d,e^	1.6 ± 0.5^e^	1.2 ± 0.6	0.8 ± 0.3	0.8 ± 0.3
Cholesterol (mmol L^−1^)	4.7 ± 0.8	4.0 ± 1.2	4.3 ± 1.0	4.0 ± 0.6	4.6 ± 0.1
LDL-C (mmol L^−1^)	2.9 ± 0.7	2.3 ± 0.7	2.6 ± 0.9	2.3 ± 0.5	2.6 ± 0.6
HDL-C (mmol L^−1^)	1.0 ± 0.2^a,c^	1.6 ± 1.0^b^	1.2 ± 0.2^a^	1.3 ± 0.3	1.6 ± 0.4
Fasting glucose (mmol L^−1^)	5.8 ± 1.3^e^	5.7 ± 1.8	5.6 ± 1.9	4.7 ± 0.4	5.3 ± 0.4

Values are mean ± SD. Letters indicate statistically significant difference from: ^a^healthy, ^b^pre-op obese, ^c^2-year post-RYGB, ^d^pre-diet obese, ^e^post-diet; *p* < 0.05.

BMI, body mass index; LDL, low density lipoprotein; HDL, high density lipoprotein.

Both treatments induced significant BW loss (average body weight loss for RYGB = 36.6 ± 11.9 kg and for diet = 24.1 ± 12.0 kg, *p* < 0.05). Importantly, BMI at the end of the respective follow-up was not significantly different between the two groups. Interestingly, there was no correlation between increased TMAO levels and the beneficial changes in lipid and glycaemic profile observed after RYGB or after diet (SOM Table 1). In line with the hypothesis of an up-regulated TMAO synthesis, the TMA/TMAO precursor^1^ choline was decreased after RYGB (Fig. 1C) and circulating bile acids (BA) (Fig. 1D), which are upstream regulators of FXR-dependent TMAO-producing FMO3^1^, were increased. Our obese rat model, in which we previously demonstrated early and sustained improved endothelial function after-RYGB^18^, recapitulated the patient scenario showing increased circulating TMAO (mean TMAO concentrations 19.08 μmol/L) and bile acids (Fig. 1E,F) and decreased choline (SOM Fig. 1C) eight days after surgery when compared to sham-operated obese rats. In the rat model, we further investigated whether enhanced BA-dependent FXR activation might mediate higher TMAO synthesis post RYGB. In rat livers, FXR protein expression was similar in obese sham-operated versus RYGB animals (Fig. 1G), however, genes under FXR control^19^ were up-regulated (i.e. Nr0b2 encoding *Shp* protein) or down-regulated (i.e. *Cyp7a1, Cyp8b1, Slc10a1 (*encoding *Ntcp), Slc1b2 (*encodin*g Oatp1b3), Slc22a1 (*encoding *Oct1)*), respectively (Fig. 1H). This gene signature^19^ reliably indicates higher BA-dependent FXR activation after RYGB compared to obese sham-operated controls (Fig. 1H,I). Of note, we measured TMAO circulating concentrations also in obese sham-operated versus RYGB rats 1 month after intervention and confirmed higher TMAO levels after bariatric surgery (mean TMAO concentrations 1.12 and 5.13 μmol/L, respectively) (SOM Fig. 1D).

### Acute TMAO administration does not affect vasodilatatory function in rat

**Figure 2:**
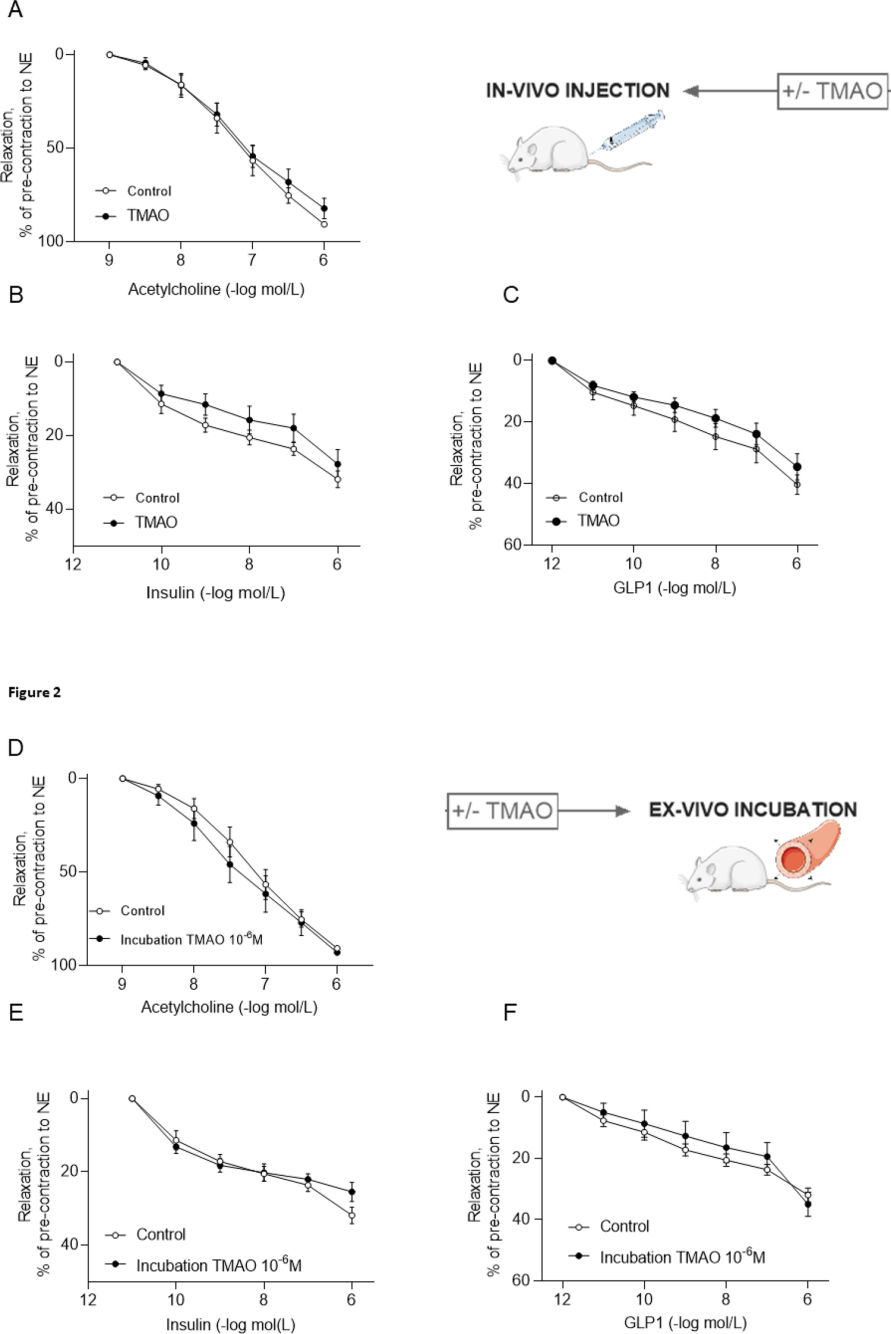
Acute TMAO administration in rats does not impair aortic endothelial function. In rats injected with TMAO (7 mg kg^−1^ i.p.) 1.5 h before sacrifice, vasodilatation elicited by (**A**) acetylcholine (**B**) insulin and (**C**) GLP-1 was similar to control animals. Similarly, vasodilation in response to (**D**) acetylcholine, (**E**) insulin, or (**F**) GLP-1 of rat thoracic aortic rings pre-incubated ex-vivo for 30 min with 10^−6^ M TMAO remained unchanged. Mixed-effect analysis or 2-way ANOVA, n = 5–6 rats. TMAO: trimethylamine N-oxide, GLP-1: glucagon-like peptide-1, eNOS: endothelial nitric oxide synthase, NO: nitric oxide, NE: norepinephrine.

To explore the vascular specific effects of TMAO, we used a combination of in-vivo and ex-vivo investigations in metabolically healthy lean rodents. Aortic vasodilation in response to Ach in rats injected i.p. with TMAO (7 mg kg^−1^) 1.5-h before sacrifice was similar to vehicle-treated rats (Fig. 2A). There was also no difference in vasodilation between the two rat groups in response to partial-endothelial agonists, such as insulin and glucagon-like peptide-1 (GLP-1) (Fig. 2B,C), which can more sensibly detect early and subtle endothelial dysfunction^18^. We also assessed effects on vasorelaxation after 30-min ex-vivo pre-incubation of aortas with TMAO. Again, we did not see any difference in vasodilation in response to Ach, insulin and GLP-1 in the presence or absence of TMAO-pre-incubation at physiological concentrations (10^−6^ M) that resemble circulating levels measured in humans and rats after bariatric surgery and atherosclerosis (Fig. 2D–F) or at the pharmacological high concentration of 10^−4^ M (SOM Fig. 2A–C). Therefore, the acute administration of TMAO did not affect vasodilation of rat thoracic aorta.

### Acute TMAO treatment does not affect stroke size or thrombus formation in mice

**Figure 3:**
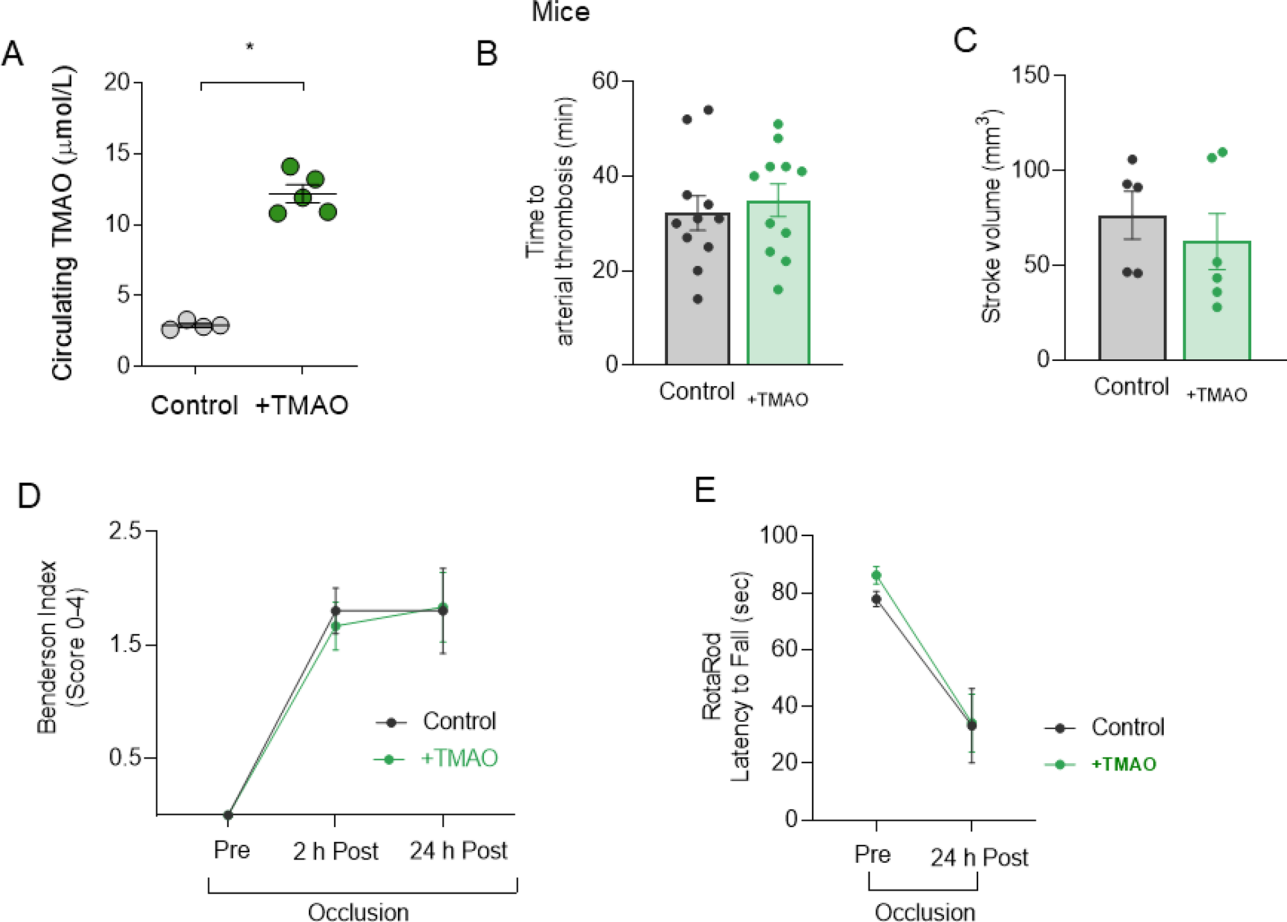
TMAO in-vivo treatment in mice does not worsen arterial thrombosis or stroke outcome. In mice, (**A**) mean circulating TMAO concentrations one hour after i.p. injection. (**B**) Time to arterial thrombosis and (**C**) stroke volume were unchanged in TMAO pre-treated compared to control animals. (**D**) Neurological recovery after stroke and (**E**) motor recovery show no difference between mice groups. Unpaired t-test or 1-way ANOVA with Dunnett Correction where appropriate, n = 5–11 mice in (**B**–**E**). TMAO: trimethylamine N-oxide. For TMAO measurement, in (**A**), n = 4 PBS-treated controls and n = 5 TMAO-treated mice.

As TMAO was associated with enhanced platelet reactivity and thrombogenesis^6,20^, which are pivotal players in arterial thrombosis and ischemic stroke, we assessed the effects of acute TMAO treatment on arterial thrombus formation and ischemic stroke in lean wild-type male mice. Notably, the serum concentrations obtained in mice after TMAO injection were in the lower µM range and closely mimicked circulating levels measured in obesity and after RYGB (mean TMAO concentrations 12.18 vs 2.90 μmol/L, one hour after TMAO i.p. injection or vehicle, respectively, *p* < 0.05, Fig. 3A, n = 5 TMAO-treated mice and n = 4 PBS-treated controls). The time to arterial occlusion did not differ between TMAO-treated and control mice (Fig. 3B), suggesting that TMAO did not acutely alter thrombogenicity in our model of carotid artery thrombosis following endothelial-specific injury. Similarly, 24-h after transient middle cerebral artery occlusion (t-MCAO, a model of ischemia–reperfusion cerebral injury), animals pre-treated with TMAO showed similar cerebral infarct sizes compared to controls (Fig. 3C). Coherently, treated and control animals did not differ in terms of post-stroke neuromotor deficit as assessed by both Bederson score (Fig. 3D) or RotaRod test (Fig. 3E). In sum, acute TMAO treatment in healthy mice did not worsen arterial thrombus formation or ischemic stroke.

### Acute TMAO treatment does not influence endothelial nitric oxide production and stress-activated signalling

**Figure 4:**
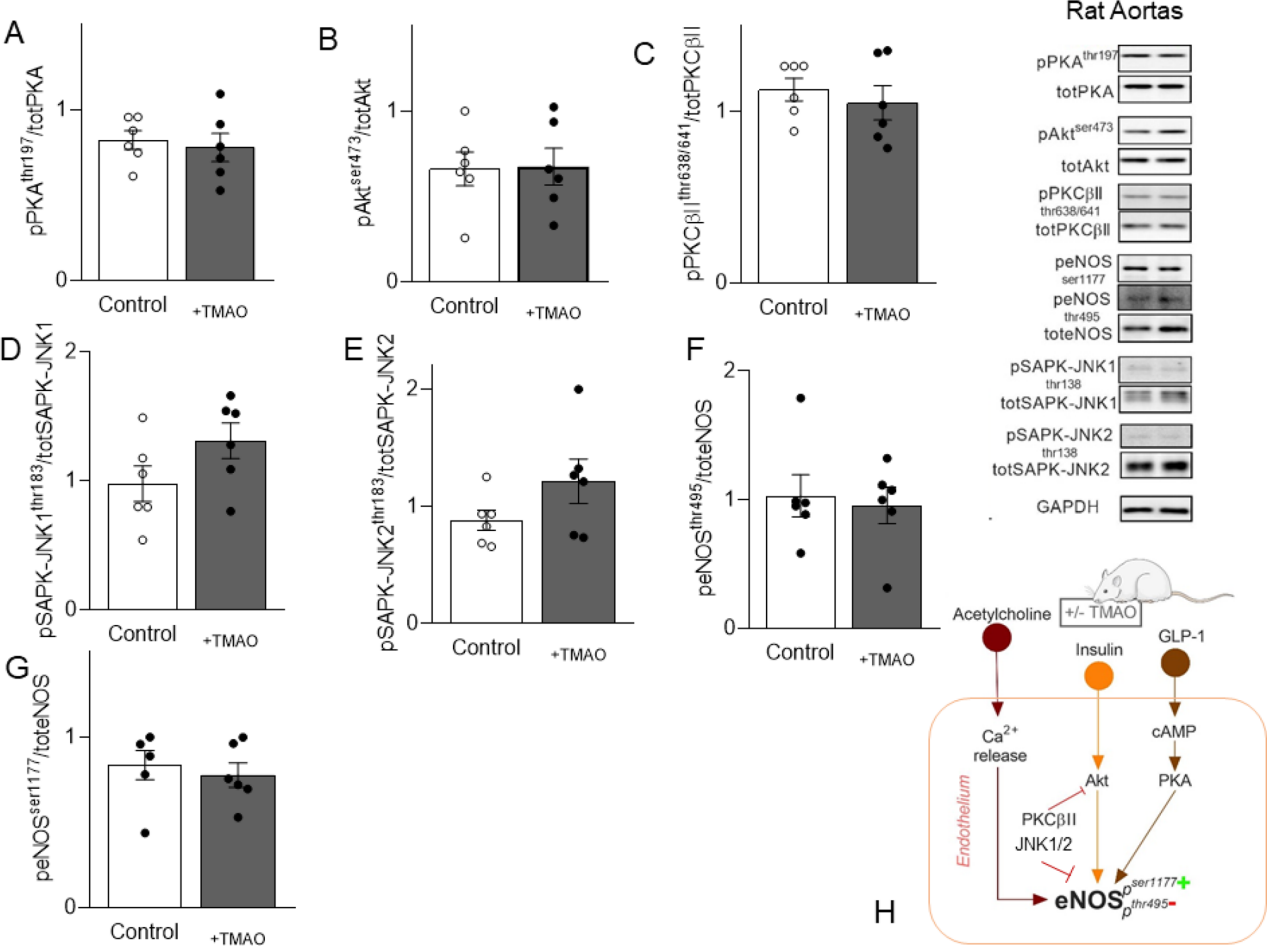
TMAO injection does not alter rat aortic eNOS-NO molecular signalling. In the aorta of rats treated with TMAO before sacrifice, key vasodilatory signalling and stress-induced signalling were unchanged. (**A**) PKA phosphorylation, (**B**) Akt phosphorylation, (**C**) PKCbII phosphorylation, (**D**) SAPK-JNK1 and (**E**). SAPK-JNK2 phosphorylations are not affected. (**F**) eNOS threonine 495 inhibitory phosphorylation and (**G**) eNOS serine 1177 activatory phosphorylation. (**H**) Diagram of the addressed eNOS-NO signalling. Rat data: Original blots/gels are presented in Supplementary Fig. 4. Unpaired T-test, n = 6 in each group. TMAO: trimethylamine N-oxide, GLP-1: glucagon-like peptide-1, eNOS: endothelial nitric oxide synthase, PKA: protein kinase A, Akt: protein kinase B, PKCßII: protein kinase C ßII, SAPK-JNK: stress activated protein kinases/Jun-amino terminal kinases.

**Figure 5:**
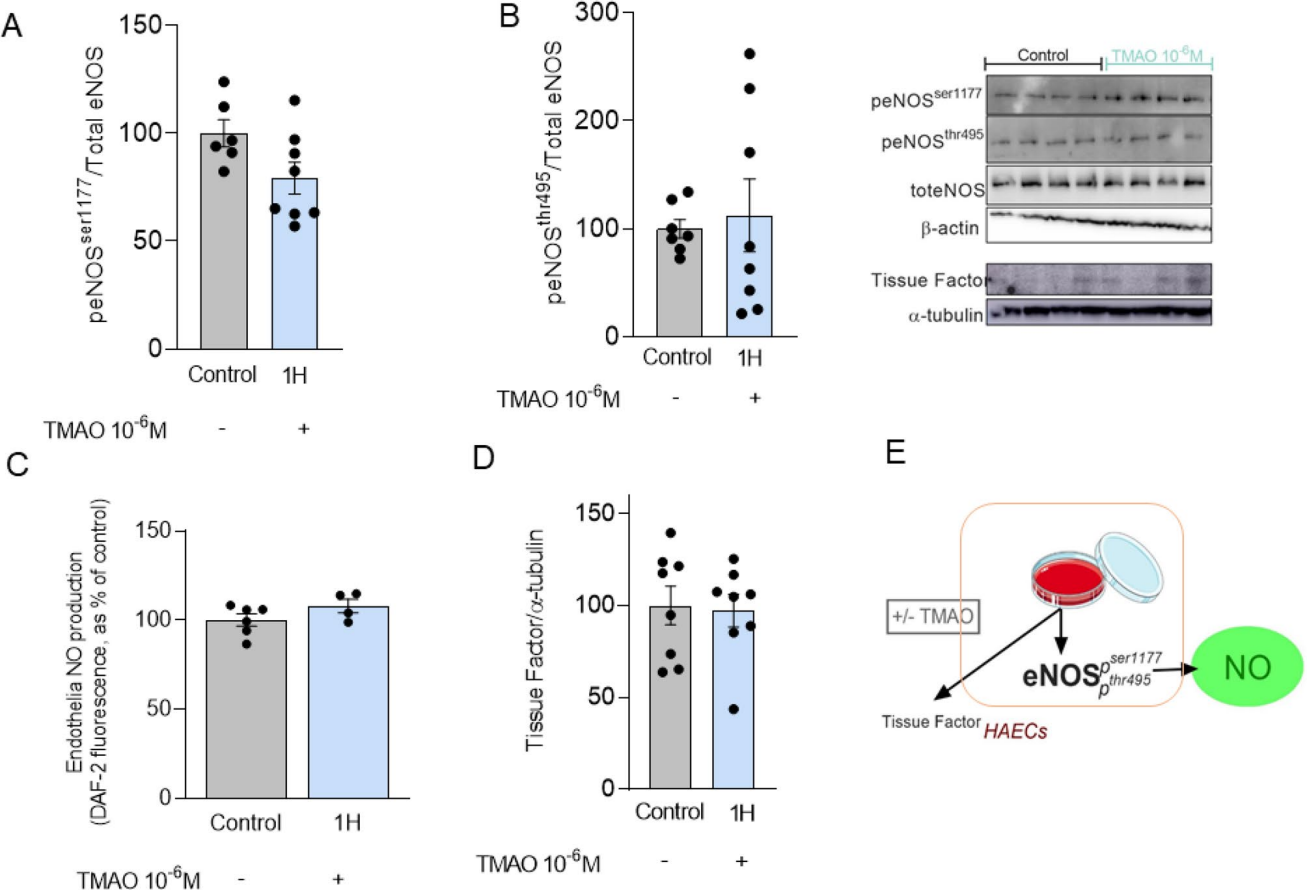
Acute administration of TMAO does not impair NO production. In HAEC incubated with TMAO (10^−6^ M) for one hour, Western Blot protein expression revealed no differences in eNOS phosphorylation (**A**) at the activatory site, serine 1177, and (**B**) the inhibitory site, threonine 495. Similar was also the (**C**) endothelial NO production. (**D**) Endothelial tissue factor expression was not induced by TMAO incubation. (**E**) Summarizes the observed in-vitro effects of TMAO incubation in HAECs. Original blots/gels are presented in Supplementary Fig. 5. Unpaired T-test or 1-way ANOVA with Dunnett correction, n = 4independent cell-culture experiments with 1–2 replicates per experiment per condition. TMAO: trimethylamine N-oxide, HAECs: human aortic endothelial cells, eNOS: endothelial nitric oxide synthase, NO: nitric oxide.

Ach, insulin and GLP-1-dependent endothelial nitric oxide synthase (eNOS) activation produces NO, which in turn induces vasodilatation^18^. Thus, we analysed PKA, Akt and PKCßII by Western Blot in aortas of TMAO-treated rats, which are key secondary messengers modulating eNOS activation (Fig. 4A–C). Phosphorylation and total protein expression of these targets as well as the stress activated protein kinase/Jun-amino terminal kinase (SAPK-JNK) isoform 1 and 2, were not significantly altered (Fig. 4D,E). Similarly, eNOS inhibitory (Thr495) and activatory (Ser1177) phosphorylation (Fig. 4F–H) were not modified by TMAO treatment. Further, we incubated human aortic endothelial cells (HAEC) with TMAO at physiological concentrations of 10^−6^ M (Fig. 5) or at the pharmacological high concentrations of 10^−4^ M (SOM Fig. 3). One hour incubation of HAEC with TMAO 10^−6^ M showed that eNOS activatory (Ser1177) and inhibitory (Thr495) phosphorylation (Fig. 5A,B) as well as endothelial NO production (Fig. 5C) were unchanged compared to DMSO-treated control HAECs, recapitulating observations in rat aortas. Of note, endothelial NO production was also not diminished in respect to control cells after incubation of HAEC with TMAO 10^−6^ M up to two hours (SOM Fig. 3A). To mimic in-vitro responses observed in the organ chamber, where we stimulated Ach-dependent vasorelaxation in the presence or absence of TMAO pre-incubation, HAECs were pre-incubated for one hour with TMAO (10^−6^ M) followed by one hour stimulation with Ach (10^−6^ M). As a result, Ach-induced endothelial NO production was not impaired by the pre-incubation with TMAO (SOM Fig. 3B). Similar results were observed when insulin or GLP-1 were used to stimulate endothelial NO production (SOM Fig. 3B). Of note, when HAEC were incubated with TMAO at the pharmacological concentrations of 10^−4^ M, we observed a decrease of the pSer1177 activatory and an increase of the pThr495 inhibitory phosphorylation (SOM Fig. 3C,D), although endothelial NO production (SOM Fig. 3E) was not impaired compared to control HAECs. Interestingly, tissue factor expression (Fig. 5D), a well-established clot-activating transmembrane receptor, which is upregulated in stroke, was unchanged by acute in-vitro TMAO incubation with 10^−6^ M, but significantly increased when TMAO was added at 10^−4^ M (SOM Fig. 3F). Thus, the main molecular targets leading to NO production, stress-activated intra-cellular signalling and thrombosis were not affected by acute TMAO exposure at physiological concentrations measured after bariatric surgery. TMAO exposure showed potentially negative effects at higher pathological concentrations, even if acutely the functional consequences on NO production were not detectable.

## Discussion

This paper investigates whether acute TMAO administration is associated with endothelial dysfunction or enhanced carotid thrombotic activity and stroke size using a combination of in vivo and in vitro methodologies. We translationally demonstrate that: (1) circulating TMAO plasma levels increased post-RYGB surgery in patients and in rats in parallel with sustained BW loss, and improved the gluco-lipid profile and endothelial function. (2) Acute TMAO injection at physiological levels was not associated with changes in endothelial vasorelaxation, arterial thrombogenicity or ischemic stroke severity in lean healthy rats and mice. (3) Acute incubation of rat aortas and HAEC with TMAO mimicking the plasma TMAO concentrations seen post-RYGB was not associated with changes in molecular targets of endothelial eNOS-NO pathway or stress-activated pro-thrombotic pathways. (4) Only pharmacological high concentrations of TMAO induced changes on eNOS phosphorylation status, which may decrease NO bioavailability. Taken together, these data indicate that TMAO per se does not alter directly and acutely endothelial NO bioavailability, arterial thrombogenicity and stroke size. Our results suggest that the interplay between TMAO and the underlying healthy or diseased metabolic constellation is a key determinant of the cardiovascular effects associated to this gut metabolite. Thus, we add to the complexity of the ongoing elucidation of the role of TMAO in cardiovascular disease.

RYGB substantially improves or even remits several cardio-metabolic comorbidities associated with obesity such as type 2 diabetes mellitus (T2D), dyslipidemia and hypertension, decreasing cardiovascular mortality^15^ and increasing life expectancy compared to non-surgical obesity management^21^. We previously reported the early restoration of HDL-mediated vaso-protection and endothelial function along with improved T2D and dyslipidemia in patients and rodents post-RYGB^18^. We now show that fasting circulating TMAO levels increased concomitantly with the sustained cardio-metabolic benefits in the same patients, who were followed up to two-years after RYGB. Initial observations proposed TMAO as a causal pathogenetic player of athero-thrombotic cardiovascular disease, in particular as a predictor of coronary artery disease burden and of major adverse cardiovascular events, including stroke^3,22^. Recent evidence instead suggests that plasmatic TMAO may simply reflect a dysbiotic gut microbiota, which is often associated with metabolic disarrangements and atherosclerotic CVD^23^. These diseases in fact can heavily impact the quantity and quality of the gut microbiota and the microbiota-dependent processing of dietary components^24^. Accordingly, increased circulating levels of TMAO have been associated with glucose intolerance, T2D, NAFLD and obesity in humans and mice^25^. It is however important to note that circulating TMAO levels have been correlated with obesity and NAFLD only in the presence of concomitant T2D, whereas this association was lost after adjusting for HOMA2-IR in patients without T2D^26^. Along with this notion, increased plasmatic TMAO levels measured in patients with cardiovascular disease may be the epiphenomenon of deleterious effects of the interplay between insulin resistance and systemic inflammation on gut microbiota developing as consequence of the accompanying metabolic comorbidities of these patients. Once dysregulated, the TMA/TMAO axis will then contribute in a vicious circle to further impair glucose tolerance, dysregulate hepatic insulin signalling, and promote adipose tissue inflammation favouring the progression of atherosclerosis^27^. Of note, our patients were severely obese, but had a relatively healthy accompanying metabolic profile with only slightly increased pre-operative fasting glucose levels (Table 1). Only four individuals had T2D at baseline, while at the 2 year-follow-up only one patient remained diabetic. Pre-operative lipid profile was within normal range, except for low HDL-C concentrations, which were improved post RYGB (Table 1).

In rats, we showed that RYGB increases circulating bile acid, which in turn activate hepatic FXR leading to higher plasmatic TMAO (Fig. 1H,I). Emerging understanding consider bile acids as key endogenous signaling molecules linking metabolism with gut microbiota^28^. In particular, increased circulating bile acids play a key role in BW loss along with remarkable improvements of glucose and lipid control after RYGB^29,30^. Thus, in our obese rat and human cohort, the higher TMAO concentrations after RYGB may be the epiphenomenon of a modified gut microbiota and BA-dependent hepatic FXR activation secondary to the surgical gut manipulation. Accordingly, it has been speculated that increased levels of TMAO after RYGB may occur due to the altered delivery of unabsorbed precursors due to post-operative dietary changes combined with surgical shortening of the small bowel, which creates a less anaerobic environment and thus a remodeling of the microbiome favoring TMAO-production^14^.

TMAO levels and BA concentrations did not correlate in our bariatric cohort, as previously shown in obese patients, likely because of the low sample size at the 2 year-follow-up, being a limitation of the study (SOM Table 1).

Reduced renal clearance can elevate TMAO levels^31^; however eGFR, was normal in patients after RYGB and we did not observe differences in rat kidney organic cation transporter (Oct) 1 and 2 mRNA expression, which are key transporters for TMAO excretion in rodents(data not shown)^32,33^. In our study we, like others^34^, show that TMAO levels are not modified after significant diet-induced weight loss, despite an effective improvement of cardiometabolic risk parameters (Fig. 1 and Table 1). A pronounced decrease of TMAO levels after diet modifications has been observed following the implementation of the Mediterranean diet in healthy individuals^35^, which supports the role of the underlying metabolic phenotype (i.e. health versus obesity) as a key factor modulating TMAO levels.

We did not observe sex-dependent differences in the TMAO concentration in healthy and obese patients before and after RYGB or diet-treatment (SOM 1A,B), similar to previous reports performed in healthy as well as in overweight/obese, pre-diabetic or CVD, stroke patients^1,34,36,37^. On the contrary, some studies measured higher TMAO levels in healthy men^35^, while in mice, higher circulating TMAO levels were observed in female animals^1,2^.

We confirmed a high variability in circulating fasting TMAO levels showing a large overlap between patients and healthy controls. Since a clear definition of physiological TMAO levels is missing, there are large differences across individual studies, which make difficult to identify thresholds to distinguish TMAO levels in health and disease states^10,37^. In this study, TMAO was tested in serum, in which concentrations of TMAO are more stable compared to citrate plasma^38^. In fact, different type of blood substrates and sampling practices have been implicated in the very high inter-study variability of TMAO levels^38^. Additional variability in TMAO plasmatic concentrations should also be considered in relation to intra-individual factors for instance the hormonal cycle, since transient trimethylaminuria has been reported in healthy women in relation to the menstruation^39^.

The broad overlap of TMAO concentrations between health and diseases poses some concerns about the role of TMAO as causal player in atherosclerotic CVD or when interpreting TMAO levels as a prognostic marker for incident cardiovascular events^40^. In this study, TMAO circulating levels in healthy subjects were similar to those measured in severely obese candidate to RYGB or diet treatment (Fig. 1A,B). We measured TMAO in the low µM range in obesity and post-RYGB (Fig. 1A), which are in line with concentrations reported in other RYGB cohorts^14^ and comparable to TMAO levels reported in patients with T2D, chronic kidney disease^31^, acute coronary syndrome^40^, cerebrovascular disease and in healthy individuals^11^.

For the in vivo and in vitro experiments performed in this study, we explored concentrations of TMAO in the lower µM range to closely mimic circulating levels measured in obesity and after RYGB and overall mirror clinically relevant scenarios^31,37^. We found that TMAO has no acute and direct detrimental effect on aortic relaxation in response to Ach, insulin and GLP-1 and does not alter the eNOS-NO intracellular signalling in aortas from lean rats injected with TMAO 1.5-h before sacrifice. Others have also seen no differences in Ach-dependent vasorelaxation with 60 min pre-incubation with TMAO 300 μmol/L in mesenteric arteries from lean Wistar rats and observed mild impairment in rat femoral arteries likely involving the endothelium-derived hyperpolarizing factor, although molecular mechanisms upon TMAO exposure were not elucidated^41^. In line with our experimental findings, in arterioles isolated from adipose tissue of healthy volunteers the exposure to a slightly higher concentration of 10 µmol/L TMAO for 4 h did not impact endothelium-dependent vasodilation to Ach^42^.

Similarly, studies performed in endothelial cell-culture report deleterious effects of TMAO, which is used at concentrations much higher than 10^−6^ M (usually above 10^−4^ M and even in the millimolar range). Those results assess pharmacological or overt toxic effects of TMAO rather than mimicking relevant concentrations measured in diseases, as often wrongly claimed. Notably, sustained high TMAO in serum in the mM range resulted in 50% mortality in mice^43^.

In HAEC, TMAO 100 μmol/L for 40 min triggered NFkB-dependent inflammation and enhanced endothelial recruitment of leukocytes^44^. Incubation with TMAO 300 μmol/L for 60 min increased mouse endothelial cell permeability impairing the expressions of tight junction proteins and activated TLR4- dependent inflammatory pathways^7^. However, deleterious endothelial effects were not observed when TMAO was used at either 10 or 30 μmol/L^7^. These concentrations resemble better plasmatic values observed in our RYGB rat model (19.08 and 5.13 μmol/L, average TMAO concentrations at 8 days and one month post RYGB, respectively) and in patients (6.50 vs 3.13 μmol/L post RYGB vs basal TMAO levels). Other studies in HUVEC used TMAO at 300 μmol/L for 6 h to trigger oxidative stress and impair the eNOS-NO axis^45^. Interestingly, in HAEC after one hour stimulation with TMAO at 10^−4^ M, we saw a decrease of the eNOS pSer1177 activatory and an increase of the pThr495 inhibitory phosphorylation, although endothelial NO production was still preserved (SOM Fig. 3C,D). Conceivably, this initial sign of eNOS dysfunction may become visible after several hours of TMAO pharmacological high dose-mediated endothelial insult.

We also focused on the acute setting rather than on chronic TMAO effects, as in other studies^8,20,43^. These factors may explain the apparent discrepancy between our neutral effects compared to evidence of a TMAO-dependent endothelial damaging action^13,33,39^. In mouse carotid endothelial cells, NRLP3-dependent inflammatory response and loss of endothelial barrier function were observed in vitro upon treatment with TMAO at 30 μM overnight, while in vivo similar findings were recapitulated by over 14 days of TMAO infusion, at a dosage unfortunately non-specified^8^. Seldin et al.^44^ observed activation of inflammatory signalling in atheroprone mice aortas collected 30 min after TMAO i.p. injection (86 μmol) producing circulating levels around 100 μmol/L over the first hour. Our dose of 7 mg/kg corresponds to about ¼ of the dose used by Seldin et al.^44^ and produced in mice mean circulating TMAO levels of 12.18 μmol/L over the first hour (Fig. 3A).

Ischemic stroke is a heterogeneous disease with variable pathogenesis. It is currently debated whether TMAO is really an atherogenic factor or whether variations in its concentrations just reflect the presence of cardiovascular and metabolic risk factors associated with ischemic stroke^37^. Patients with recurrent stroke displayed higher plasma TMAO levels than those without recurrent stroke at 1-year follow-up (recurrent stroke TMAO average: 6.86 μM)^22^. Interestingly, patients in our study had very similar circulating TMAO of 6.50 μmol/L 2-years after RYGB, which is known to reduce major adverse cardiovascular events including ischemic stroke^46^.

In our in vivo experiments, TMAO did not acutely alter either carotid time to thrombotic occlusion or cerebral infarct sizes compared to controls. Accordingly, tissue factor endothelial protein expression was unchanged by one hour in-vitro TMAO incubation with 10^−6^ M in HAEC. Witkowski et al.^20^. observed increased expression of tissue factor and VCAM1 using human microvascular endothelial cells incubated with TMAO 200 μmol/L for 2 or 6 h. In HAEC, we also observed significantly increased tissue factor protein expression when TMAO was added for one hour at 10^−4^ M (SOM Fig. 3F) confirming that pharmacological concentrations of TMAO may alter the homeostatic endothelial function. Further in their study, C57/BL6 mice receiving TMAO i.p. 1.5 h before testing had enhanced tissue factor and VCAM1 expression in aortas^20^. Unfortunately, we did not assess aortic pro-inflammatory signalling under in vivo conditions, being a limitation of the study.

### Limitations

Our study is limited to a short-term TMAO exposure in healthy rodent models as attempt to dissect direct and immediate mechanistic TMAO effects on the vasculature with focus on endothelial function. Although we followed up a rather small cohort of patients undergoing RYGB, our and other observations^14^ of increased plasmatic TMAO post-RYGB indicates that there is a complex, multi-organ crosstalk involved in TMAO signalling. Unfortunately, vascular endothelial function in patients before and after Roux-en-Y was not measured in this study to assess the impact of changes in TMAO levels. Similarly, fecal samples for gut microbiota analysis were not available in our patients, limiting the possibility to dissect further the role of gut microbiome in relationship with TMA and bile acids (especially secondary bile acids).

## Conclusion

**Figure 6:**
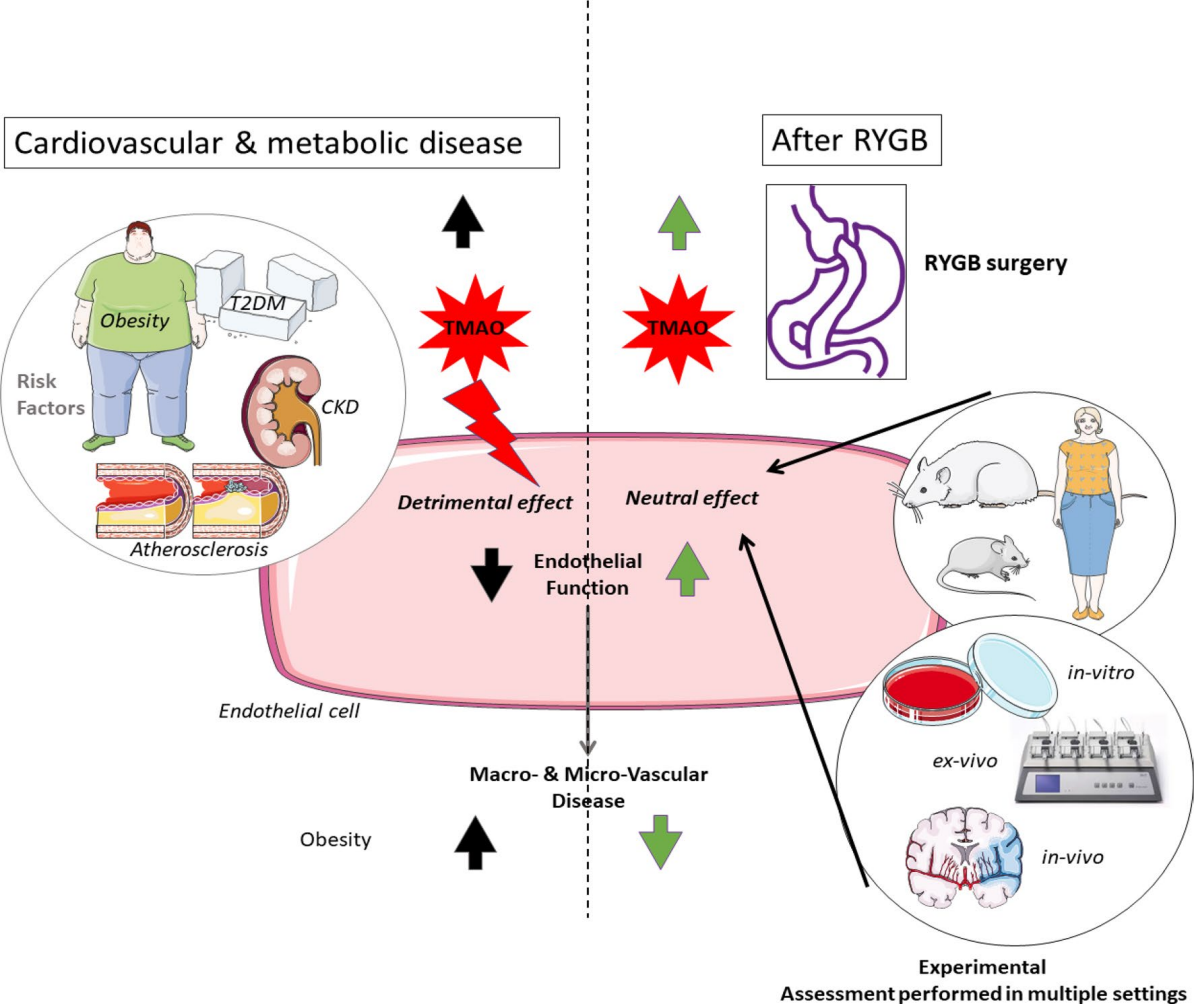
Graphical Abstract. A significant and stable increase in circulating TMAO occurs after RYGB, which is known to decrease cardiovascular morbidity and mortality. In healthy rodent models in-vivo and in-vitro, we showed that acute exposure of aortas and endothelial cells to TMAO is functionally neutral at concentrations similar to those reported in patients and rodent models post-RYGB. These findings may add a different perspective on the current understanding of TMAO as deleterious molecule promoting endothelial dysfunction and atherosclerosis.

Our results indicate that increased plasmatic TMAO in the near-physiological range may be a neutral bystander to vascular function as seen in patients after bariatric surgery or in healthy lean rodent models and in endothelial cells exposed acutely to TMAO. Therefore, the role exerted by TMAO in the development of cardiovascular disease seems to be modulated by multifactorial metabolic constellations, as graphically summarized in Fig. 6. Further research should dissect the role of TMAO in health and disease taking carefully into account the complex relationship between the cardiovascular and metabolic systems, to understand how these key players, are interacting to promote whole-body health or disease.

## Material and methods

### Patient studies

The surgery group consisted of 29 patients undergoing primary laparoscopic proximal RYGB. The diet group consisted of 21 obese patients matched for BMI, age and sex to the RYGB group 2 years after surgery (n = 14). Moreover, 33 normal-weight volunteers age- and sex-matched were enrolled.

The local Research and Ethic Committees in Zurich, Switzerland and in Padova, Italy approved the study. All patients gave written informed consent. Studies were performed according to the principles of the Declaration of Helsinki.

### Blood sampling

Blood samples were obtained after overnight fasting in healthy subjects or in patients before RYGB and starting of diet and at follow-up appointments. Serum was collected in non-additive vacutainers (BD, Heidelberg, Germany) that were kept at + 4 °C before and after blood collection. TMAO and choline were determined using liquid chromatography coupled to mass spectrometry, as described^47^. Bile acids were determined by enzymatic assay (Roche Cobas® Integra 8000 device). Plasmatic lipids (total cholesterol, HDL-cholesterol and triglycerides) and fasting glycemia were measured using nuclear magnetic resonance at Numares, AG using their Axinon system.

### Animal studies ethics and study design

All methods were carried out in accordance with relevant guidelines and regulations and the animal study protocol were approved by the Zurich Cantonal Veterinary office (ZH060/15 and ZH087/18, and ZH219/18). All methods are reported in accordance with ARRIVE guidelines (https://arriveguidelines.org).

### Roux-en-Y gastric bypass in rats

Male Wistar rats (Janvier, France) were fed a high-fat, high-cholesterol diet containing 60% kcal fat and 1.25% cholesterol for 7 weeks to achieve diet-induced obesity. Obese rats underwent sham (n = 12) or RYGB (n = 12) surgery and were assessed 8-days post-surgery^18^. In addition, we measured circulating TMAO also in rats, sham (n = 8) or RYGB (n = 8), one-month post-surgery, as published^18^.

### Treatment of lean rats with TMAO

For the acute TMAO injection experiment, adult male Wistar rats obtained from Janvier, France, weighed 250–300 g at arrival (approx. 12 weeks of age). Animals were randomized to either TMAO injection (n = 6) (7 mg kg^−1^) or vehicle PBS (n = 6), injected 1.5 h before sacrifice. The concentration was adapted from the first article testing^43^ the serum concentration of TMAO following its injection i.p. in mice. A single injection of 7 g/kg gave a peak serum average concentration around 50 mM in the first hours. We therefore injected in our rodent experiments a thousand times lower concentration of TMAO.

### Tension myography experiments

Whole thoracic aorta was excised and immediately placed in physiological Krebs buffer (in mmol/L: NaCl (118.6), KCl (4.7), CaCl2 (2.5), KH2PO4 (1.2), MgSO4 (1.2), NaHCO3 (25.1), glucose (11.1), calcium EDTA (0.026)) on ice, before being cut into ≈ 2 mm thick rings (8 aortic rings per each rat) and mounted on isometric force transducers (Multi-Myograph 610 M, Danish Myo Technology A/S, Aarhus, Denmark) within 1 h of sacrifice. Isometric tension was recorded continuously. After a 30-min equilibration period, rings were gradually stretched to the optimal point of their length-tension curve as determined by the contraction in response to potassium chloride (100 mmol). Concentration–response curves were obtained in a cumulative fashion. Several rings (range 2–4 rings) cut from the same artery were studied in parallel for each mediator^18^. Cumulative relaxation responses of the thoracic aorta were performed to acetylcholine (10^−12^–10^−6^ mol/L), insulin (10^−11^–10^−6^ mol/L), and GLP-1 (10^−11^–10^−6^ mol/L) after submaximal contraction with norepinephrine (10^−6^ mol/L)^18^.

### In-vivo carotid artery thrombosis model

Before proceeding with the in-vivo Carotid Artery Thrombosis Model TMAO circulating concentrations were assessed in a pilot group of n = 4 PBS-treated controls and n = 5 TMAO-treated mice receiving the treatment as described, below. TMAO was measured by liquid chromatography coupled to mass spectrometry, as described^47^.

After the pilot test, 3 months old C57BL/6 male mice (n = 5–11) underwent photochemical injury of the common carotid artery (CCA) as previously described^48^. TMAO treatment (7 mg kg^−1^) was performed by intra-peritoneal injection every 24-h starting 49-h before surgery. 1-h after the second injection, mice were anaesthetized by i.p. injection of sodium pentobarbital (Butler, Columbus, OH, USA). The depth of anaesthesia was confirmed by the absence of twitch reflex. Rose Bengal (Fischer Scientific, Fair Lawn, NJ, USA) was diluted to 12 mg/mL in phosphate-buffered saline and then injected through the tail vein at a concentration of 63 mg/kg. Mice were placed in supine position under a dissecting microscope and the right common carotid artery was exposed by a midline cervical incision. A Doppler flow probe (Model 0.5 VB, Transonic Systems, Ithaca, NY, USA) was applied and connected to a flowmeter (Model T106, Transonic Systems, Ithaca, NY, USA). Five to ten min after Rose Bengal injection, a 1.5 mW green light laser (540 nm; Melles Griot, Carlsbad, CA, USA) was applied to the site of injury at a distance of 6 cm from the artery for 60 min or until thrombosis occurred. From the onset of injury, carotid blood flow and heart rate were continuously monitored up to 120 min. Occlusion was defined as blood flow below 0.1 mL/min for at least 1 min.

### Transient middle cerebral artery occlusion (t-MCAO)

To induce I/R brain injury, tMCAO was performed as previously described in TMAO- or vehicle-treated mice^49^. TMAO treatment (7 mg kg^−1^) was performed by intra-peritoneal injection every 24-h starting 49-h before surgery. 1-h after the second injection, 3 months-old C57BL/6 male mice (n = 5–11) were anaesthetized using isoflurane 3% and 1.5% for induction and maintenance respectively. Body temperature was kept at 37 °C using a heating pad, which was controlled by an anal probe. For analgesia, 0.5% bupivacaine was infiltrated at the incision side. Ischemia was induced by inserting a 6–0 silicone-coated filament (Doccol Corporation, Sharon, MA, USA) into the common carotid artery until the origin of the left MCA after the dissection of common, internal and external carotid arteries. After 45 min, reperfusion was allowed by removing of the threat. Neurological status was assessed 2, and 24 h after t-MCAO by a four-point scale neurological score based on Bederson et al.61 as follows: grade 0, normal neurological function; grade 1, forelimb and torso flexion to the contralateral side upon lifting the animal by the tail; grade 2, circling to the contralateral side; grade 3, leaning to the contralateral side at rest; grade 4, no spontaneous motor activity, as previously described29. Neurological performance was determined by the RotaRod test 24 h after t-MCAO: animals were placed on a rotating rod at increasing speeds (4–44 revolutions/min) and latency to fall was recorded. After the experiments, animals were euthanized with carbon dioxide. For determination of stroke volumes, brains were cut into 5 (2-mm thick) coronal sections and immersed in a 2% solution of 2,3,5-triphenyltetrazolium chloride (TTC) (Sigma-Aldrich, Chemie GmbH, Buchs, Switzerland) at 37 °C for 20 min. Stroke areas were quantified using ImageJ Software (Image J, NIH, MD, USA). The following formula was applied in order to compensate for cerebral swelling (edema) and subsequent overestimation of the infarct volume: corrected infarct volume = contralateral hemisphere volume − (ipsilateral hemisphere volume − infarct volume).

### Endothelial cell in-vitro experimental model

Primary human aortic endothelial cells (HAECs) were obtained from Lonza, Basel, Switzerland, and cultured in endothelial cell basal medium-2 (Lonza) supplemented with endothelial growth medium–SingleQuots as indicated by the manufacturer (37 °C, 95% O2, 5% CO2). HAECs were grown to sub-confluency and rendered quiescent before experiments by incubation in medium containing 0.5% foetal bovine serum. Thereafter, cells were treated for 1-h with 10-4 M or 10-6 M TMAO (Sigma-Aldrich, 317594) dissolved in DMSO. In a separate experiment, cells were co-incubated with 10-6 M of acetylcholine, to account for pathway cross-interactions. A total of 4 independent cell-culture experiments were performed, with 1–2 replicates per experiment per condition. After incubation, cells were washed with ice-cold PBS, 150uL of lysis buffer (120 mmol/L sodium chloride, 50 mmol/L Tris, 20 mmol/L sodium fluoride, 1 mmol/L benzamidine, 1 mmol/L dithiothreitol, 1 mmol/L EDTA, 6 mmol/L EGTA, 15 mmol/L sodium pyrophosphate, 0.8 ug/mL leupeptin, 30 mmol/L p-nitrophenyl phosphate, 0.1 mmol/L phenylmethylsulfonyl fluoride, and 1% NP40) containing protease- and phosphatase-inhibitors was added to each well and plates were immediately frozen. We use a combination of freeze–thaw cycles and plate scratching to prepare samples for Western Blotting. Measurement of endothelial cell NO production by 4,5-diaminofluorescein diacetate (DAF-2) staining. Endothelial cell NO production was determined as described^18,50^. In brief, after 4-h starvation, HAEC were incubated for 1- or 2-h at 37 °C with DAF-2 diacetate (1 μM; Sigma-Aldrich D2813), that forms the fluorescent triazolofluorescein upon reaction with cellular NO. Following incubation, cells were transferred to a black microplate and fluorescence was measured on a Tecan Infinite M200 PRO reader (Tecan, Maennedorf, Switzerland) with excitation and emission wavelenghts of 485 nm and 535 nm, respectively.

### Tissue processing and Western Blot

Frozen aortae or frozen stimulated cells were dissolved in lysis buffer (120 mmol/L sodium chloride, 50 mmol/L Tris, 20 mmol/L sodium fluoride, 1 mmol/L benzamidine, 1 mmol/L dithiothreitol, 1 mmol/L EDTA, 6 mmol/L EGTA, 15 mmol/L sodium pyrophosphate, 0.8 ug/mL leupeptin, 30 mmol/L p-nitrophenyl phosphate, 0.1 mmol/L phenylmethylsulfonyl fluoride, and 1% NP40) for immunoblotting. The samples (30 µg) were subjected to SDS-PAGE gel for electrophoresis and incubated with SAPK/JNK and phospho-SAPK/JNK (Thr183/Tyr185) antibodies (Cell Signalling, Beverly, Massachusetts, USA), eNOS/NOS Type III, eNOS (pS1177) antibodies (BD Biosciences, San Jose, CA, USA), eNOS (pT495) (BD Biosciences), Akt and phospho-Akt (Ser473) antibodies (Cell Signalling), PKCßII and phospho-PKCßII (threonine 638/641) antibodies (Cell Signalling), PKA and phospho-PKA (threonine 197) antibodies (Cell Signalling) and anti-GAPDH antibody (Cell Signalling). The immunoreactive bands were detected by chemiluminescence (Amersham Biosciences, Buckinghamshire, UK) and quantified densitometrically with Image J software (National Institutes of Health, Bethesda, Maryland, USA).

### Isolation of total RNA and relative quantification by real time RT-PCR

Total RNA was isolated from frozen liver specimens by TRIzol method (Thermo Fisher Scientific, Carlsbad, CA) and quantified at 260 nm by Nanodrop. cDNA was synthesized from two micrograms of total RNA using oligo(dT) primers and Superscript II retrotranscriptase (Thermo Fisher Scientific, Carlsbad, CA). The cDNA was amplified by real time PCR analysis with the TaqMan Universal PCR Master Mix (Thermo Fisher Scientific, Carlsbad, CA) for the following targets: Nr0b2 (encoding Shp) (Rn00589173_m1), Cyp7a1 (Rn00564065_m1), Cyp8b1 (Rn01445029_s1), Slc10a1 (encoding Ntcp) (Rn00566894_m1), Slco1b2 (encoding Oatp1b2) (Rn01492634_m1), Slc22a1 (encoding Oct1) (Rn00562250_m1) and 18S rRNA (4318839).

### Statistics

Continuous variables were presented as mean ± SEM. Discrete variables were summarized as frequencies and percentages. The distribution of the data was analysed with a 1-sample Shapiro–Wilk test. Logarithmic transformation was performed to achieve normal distribution for skewed variables. Continuous data were compared by use of the 2-tailed paired or unpaired t test (for normally distributed data sets) or the Mann–Whitney U or Wilcoxon signed-rank test (for skewed variables). We used a 2-way ANOVA with repeated measures to compare repeated measurements on the same animals. For studying the complete outcome of each variable over time, we applied the linear mixed model using an unstructured covariance matrix for quantitative variables. All tests were 2 sided, and statistical significance was accepted if the null hypothesis could be rejected at *p* < 0.05, Dunnett corrections are applied as indicated in the figure legends. All data were analysed and graphed in GraphPad Prism 8.4.2.

## Supplementary Information

Supplementary Information.

## Data Availability

Data supporting the findings of this study are available from the corresponding author upon reasonable request.
