# Supplementary material for: Effects of acute administration of trimethylamine N-oxide on endothelial function: a translational study
Source: Sci Rep. 2022 May 23;12:8664. doi: 10.1038/s41598-022-12720-5 (PMC9127094; doi:10.1038/s41598-022-12720-5)
Supplement: Supplementary file 1 — Supplementary Information. [file 41598_2022_12720_MOESM1_ESM.docx]

**Effects of acute administration of trimethylamine N-oxide on endothelial function: a translational study**

Anne Jomard PhD^1,2^, Luca Liberale MD, PhD^3,4^, Petia Doytcheva PhD^3,5^, Martin F. Reiner MD, PhD^3^, Daniel Müller PhD^2^, Michele Visentin PhD^6^, Marco Bueter MD, PhD^7^, Thomas F. Lüscher MD^3^, Roberto Vettor MD^8^, Thomas A. Lutz DVM, PhD^5^, Giovanni G. Camici PhD^3,9^, Elena Osto MD, PhD^2,3,9*^.

*^1^Laboratory for Translational Nutrition Biology, ETH Zurich, Zurich, Switzerland; ^2^Institute of Clinical Chemistry, University Hospital Zurich, Switzerland; ^3^Centre for Molecular Cardiology, University of Zurich, Zurich, Switzerland; ^4^First Clinic of Internal Medicine, Department of Internal Medicine, University of Genoa, Genoa, Italy; ^5^ Institute of Veterinary Physiology, University of Zurich, Zurich, Switzerland; ^6^Department of Clinical Pharmacology and Toxicology, University and University Hospital Zurich, Zurich, Switzerland; ^7^ Department of Surgery and Transplantation, University Hospital Zurich, Zurich, Switzerland; ^8^Department of Medicine, University of Padova, Via Giustiniani, 2, Padua, 35128, Italy. ^9^Department of Cardiology, Heart Center, University Hospital Zurich, Zurich, Switzerland; ^*^Corresponding Author.*

**Address for Correspondence:** Elena Osto, M.D., Ph.D., FESC

University Hospital and University of Zurich

Institute of Clinical Chemistry and University Heart Center

Wagistrasse, 14 -CH-8952 Schlieren, Switzerland

Tel: +41-44-253 30 98

Email: [elena.osto@uzh.ch](mailto:elena.osto@uzh.ch)

ORCID ID: EO 0000-0001-8196-5696

**Social Media Handle:** @OSTOLab1; @eosto1

**Supplementary Online Material**

**Supplementary Figures and legends**

**
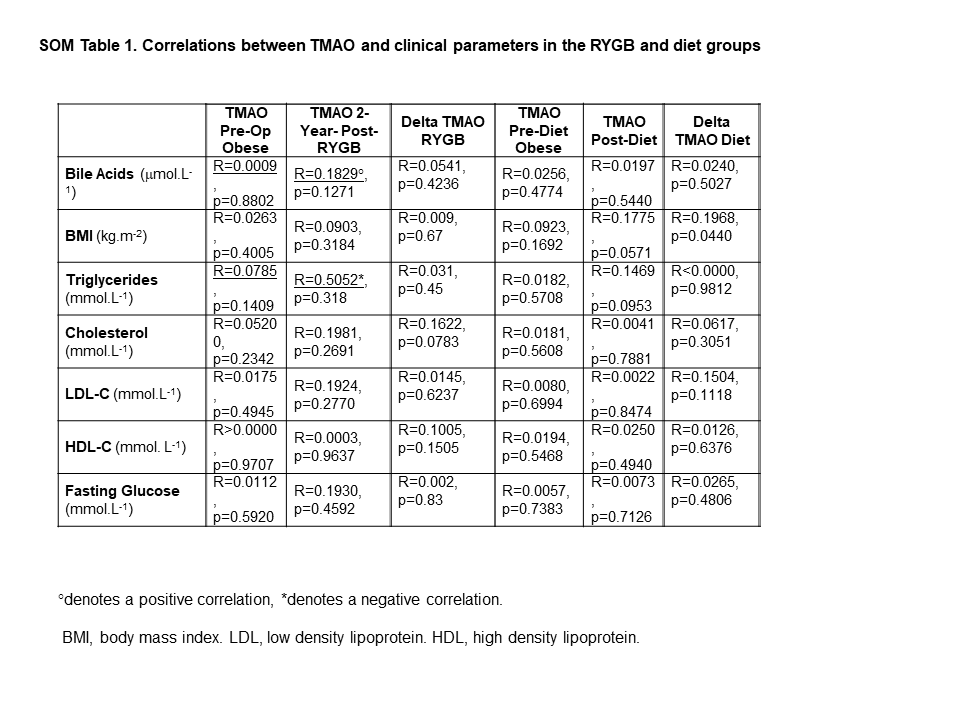

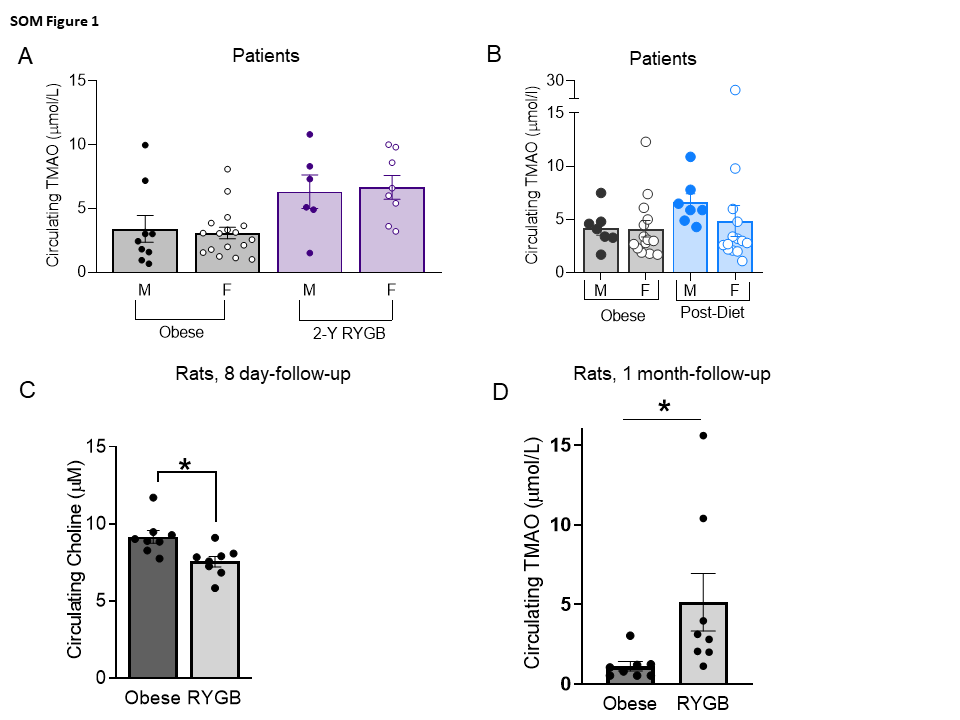
**

**SOM Figure 1. Circulating TMAO levels in patients and rats.** No sex-dependent differences in circulating TMAO levels in patients, **A.** undergoing RYGB, **B.** diet. **C.** Circulating levels of the TMAO precursor choline, in rats 8-days post-RYGB or sham-surgery (obese) and **D.** circulating levels of TMAO in rats one-month post-RYGB or sham-surgery (obese). Human data: RYGB patients n=29-14, diet group n = 21, 1-way ANOVA, Dunnett Correction. Rat data: n=5-12, Unpaired T-test. RYGB: Roux-en-Y Gastric Bypass, TMAO: trimethylamine N-oxide, TMA: trimethylamine, * P<0.05


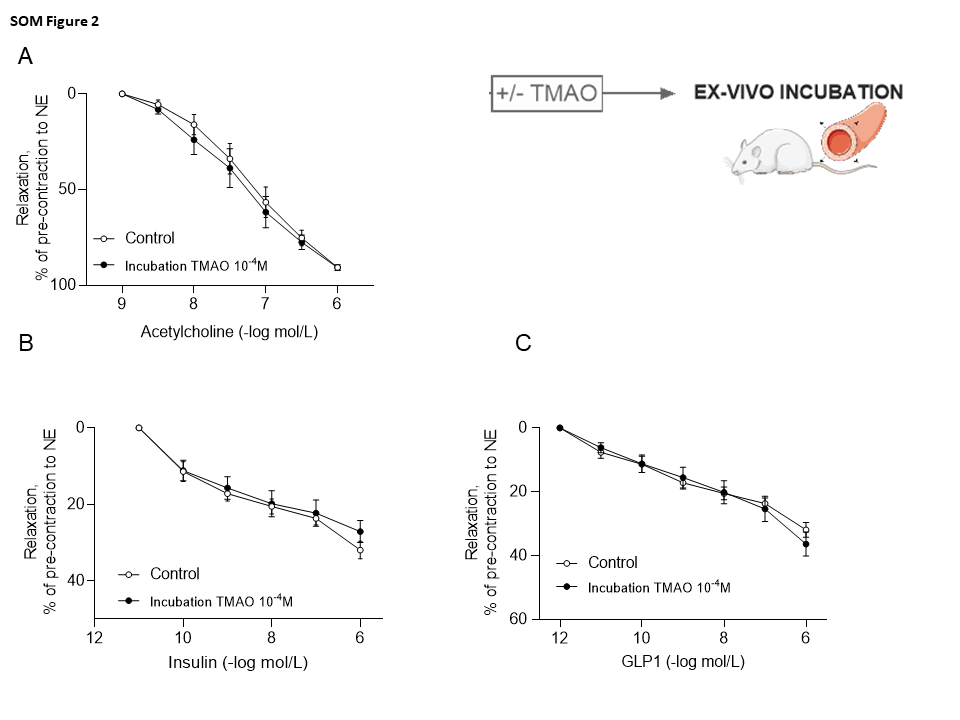


**SOM Figure 2.** **Acute TMAO *ex vivo* administration in rats does not impair aortic endothelial function.**

In rats, vasodilation in response to **A.** acetylcholine, **B.** insulin, or **C.** GLP-1 of rat thoracic aortic rings pre-incubated ex-vivo for 30 minutes with 10^-4^ M TMAO remained unchanged. Mixed-effect analysis or 2-way ANOVA, n=5-6. TMAO: trimethylamine N-oxide, GLP-1: glucagon-like peptide-1, NE: norepinephrine.


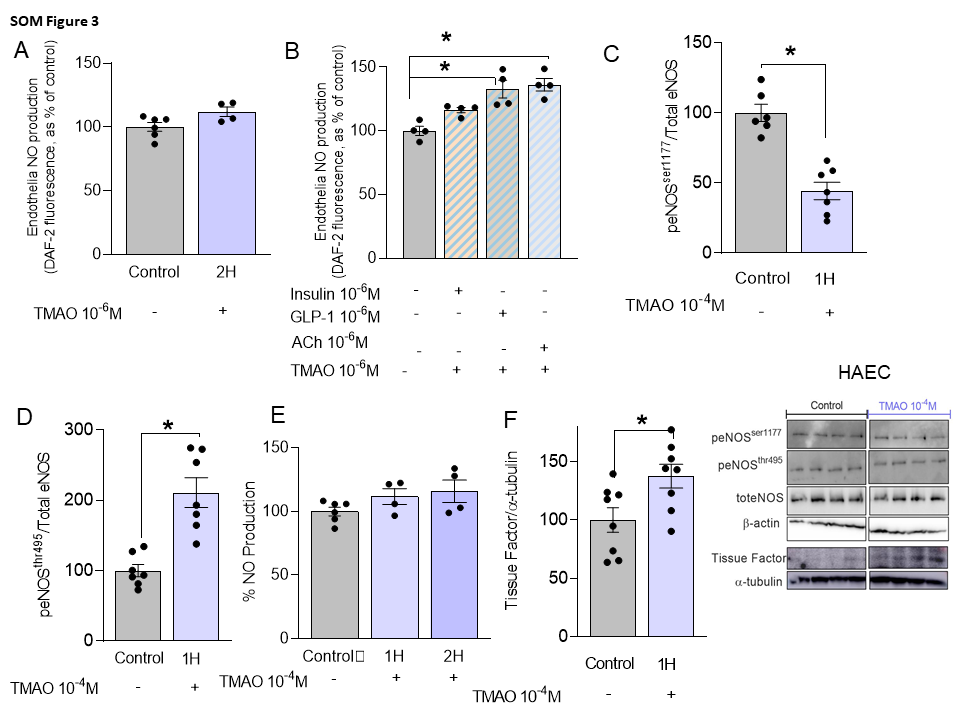


**SOM Figure 3. Acute administration of TMAO does not impair NO production.**

Endothelial NO production **A.** was not impaired in HAEC incubated with TMAO (10^-6^M) for two hours. **B.** Insulin-, GLP-1- and Ach-induced endothelial NO production was not impaired by one hour pre-incubation with TMAO (10^-6^M). Western Blot protein expression revealed in eNOS phosphorylation **C.** a decrease at the activatory site, serine 1177, and **D.** an increase at the inhibitory site, threonine 495 after one hour pre-incubation with TMAO (10^-4^M). However, **E**. Endothelial NO production was unchanged up to two hours while **F.** endothelial tissue factor expression was induced after one hour TMAO incubation (10^-4^M). Unpaired T-test or 1-way ANOVA with Dunnett correction, n=4-8. TMAO: trimethylamine N-oxide, HAECs: human aortic endothelial cells, eNOS: endothelial nitric oxide synthase, NO: nitric oxide.


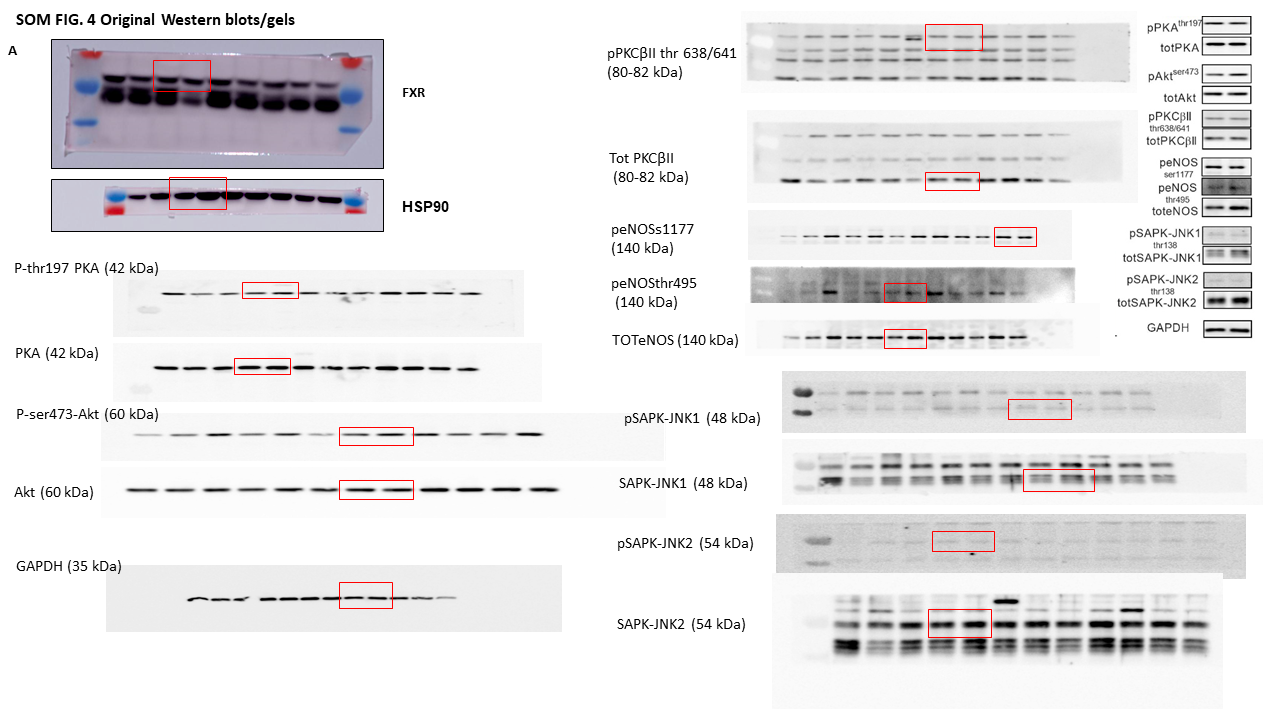


**SOM Figure 4. Original Western blots/ gels**

Original Western blots/gels **A.** of the representative images cropped in figure 1G. **B.** of the representative images cropped in figure 4.


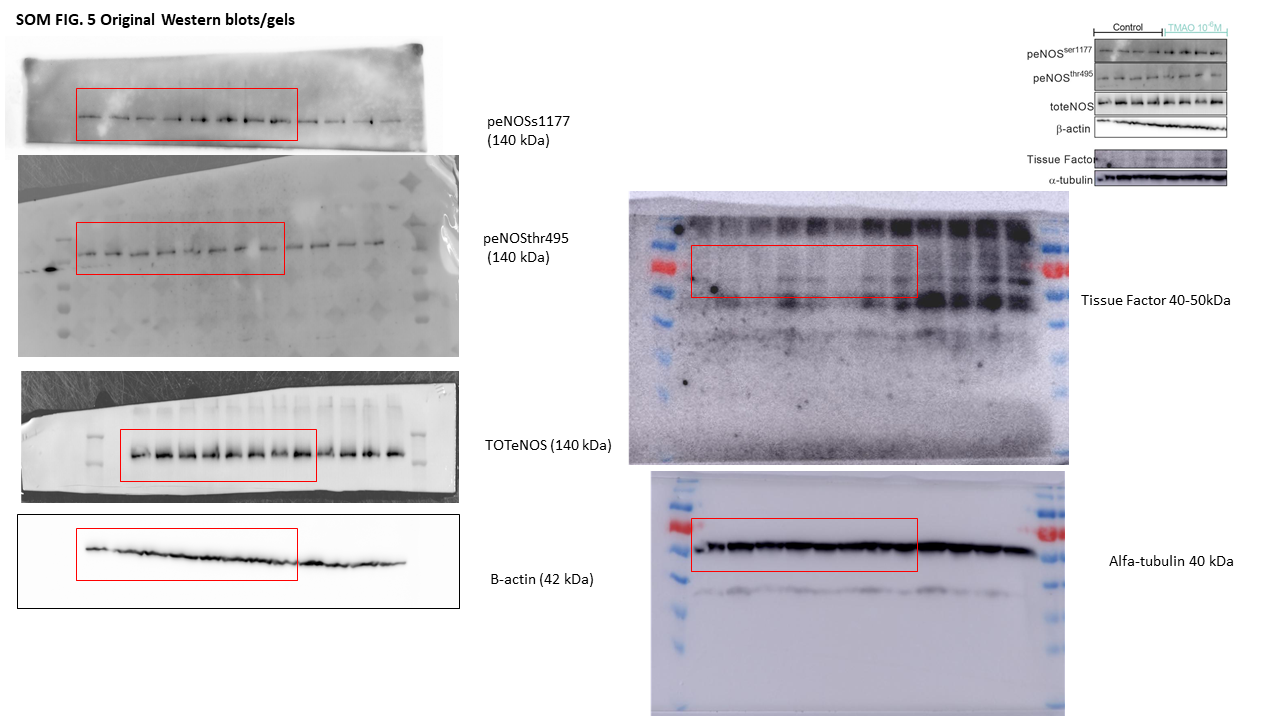


**SOM Figure 5. Original Western blots/ gels**

Original Western blots/gels A. of the representative images cropped in figure 5.


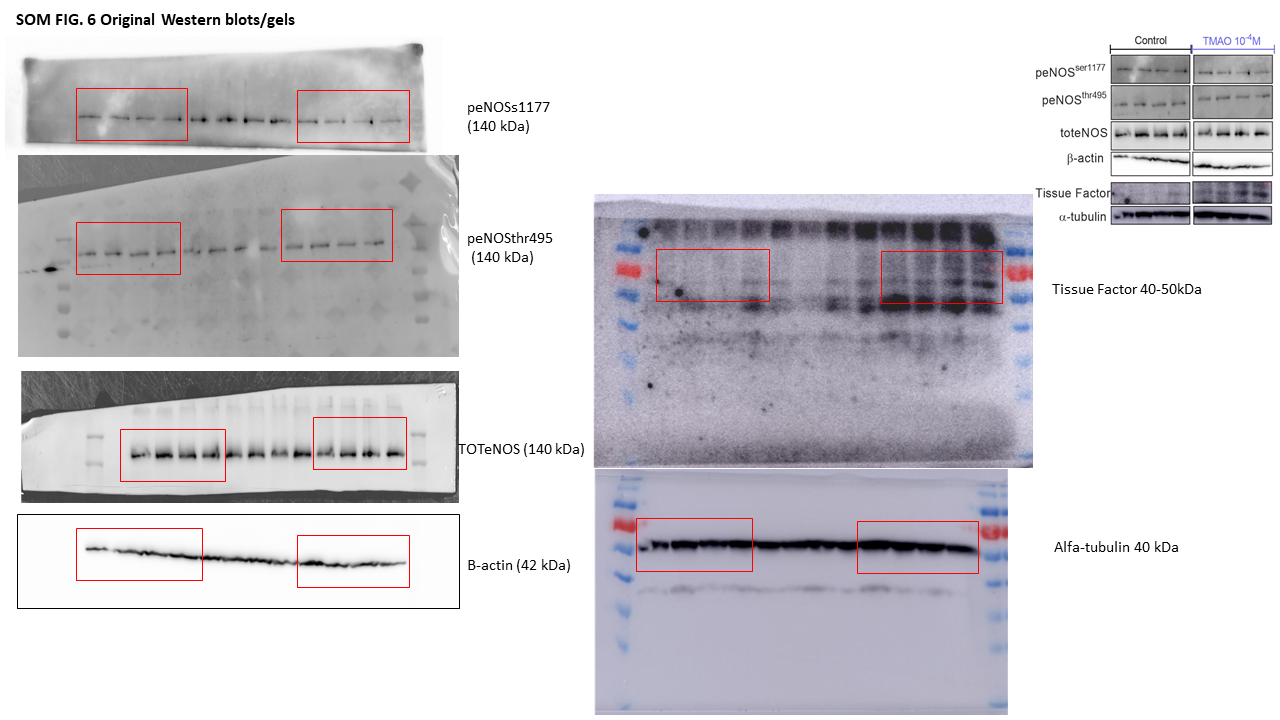


**SOM Figure 6. Original Western blots/ gels**

Original Western blots/gels A. of the representative images cropped in supplementary figure 3.


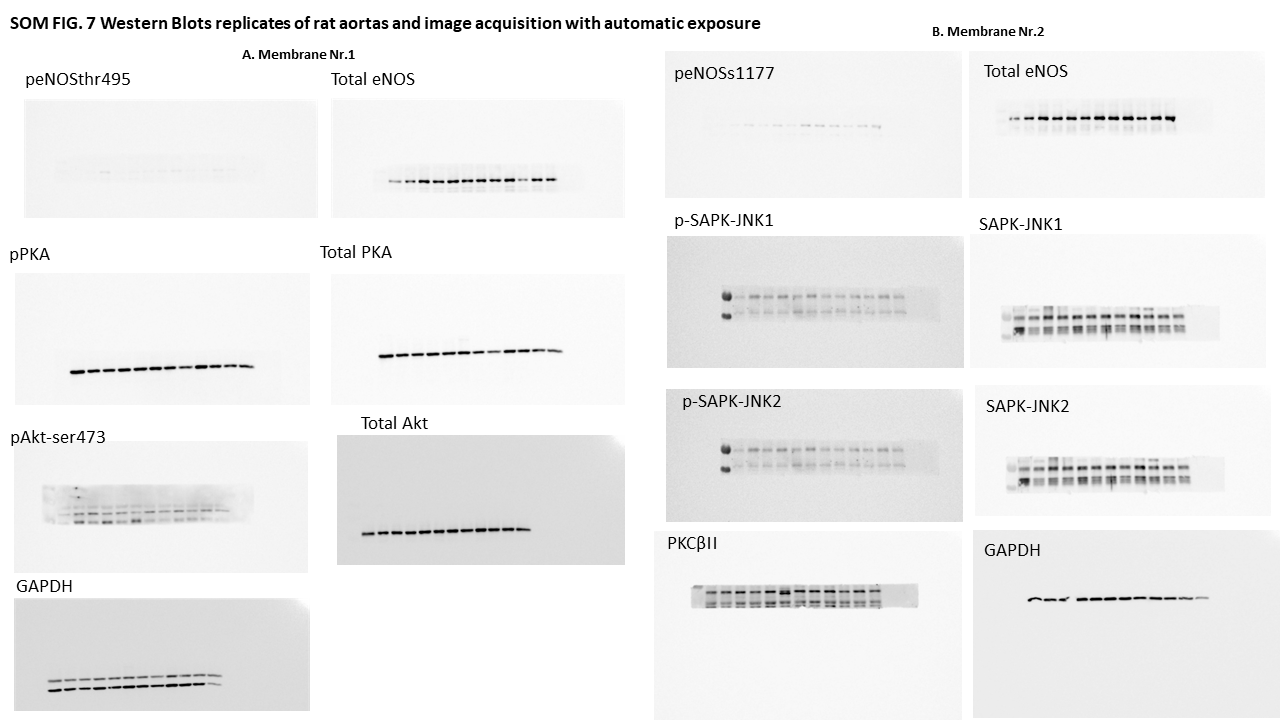


**SOM FIG. 7. Western Blots replicates of rat aortas and image acquisition with automatic exposure.**

In membrane A. Nr.1 and B. Nr. 2 the same rat aortic samples are loaded to test all different listed targets. Image acquisition was done with automatic exposure.
